# Perioperative and long-term outcomes of robot-assisted versus laparoscopy-assisted hemicolectomy for left-sided colon cancers: a retrospective study

**DOI:** 10.1007/s13304-020-00959-4

**Published:** 2021-01-04

**Authors:** Maolin Xu, Zhiming Zhao, Baoqing Jia, Rong Liu, Hongyi Liu

**Affiliations:** 1 Department of General Surgery II, The First Medical Center of Chinese, PLA General Hospital, Fuxing Road, Haidian District, Beijing, China; 2 Department of Hepatobiliary Surgery II, The First Medical Center of Chinese, PLA General Hospital, Fuxing Road, Haidian District, Beijing, China

**Keywords:** Colon cancer, Minimally invasive surgery, Da vinci robot, Laparoscopy, Survival

## Abstract

The objective of this study is to evaluate the perioperative and long-term outcomes of robot-assisted hemicolectomy (RAH) versus laparoscopy-assisted hemicolectomy (LAH) for left-sided colon cancers. Patients who underwent RAH and LAH from January 2012 to December 2018 were reviewed retrospectively. Patient characteristics and perioperative outcomes were compared between the two groups. Follow-up consultations were conducted to evaluate the long-term outcomes of these procedures. A total of 460 patients were included (RAH, *n* = 205; LAH, *n* = 255). There was no difference in patient characteristics between the two groups. Compared with the LAH group, the RAH group showed longer operative time (150.23 ± 43.77 min vs. 125.85 ± 38.67 min, *p* < 0.001) and higher surgery cost (6.33 ± 1.50 vs. 2.88 ± 0.72 thousand $, *p* < 0.001) and total hospital cost (14.97 ± 3.05 vs. 9.05 ± 2.31 thousand $, *p* < 0.001). No significant differences in tumor pathology, TNM staging, and perioperative outcomes were observed. There were no obvious differences in the 3-year and 5-year overall survival (OS) or 3-year and 5-year disease-free survival. Cox multivariate analyses showed that age, body mass index, and intravascular cancer embolus were independent risk factors for OS. Moreover, the robotic approach was not an independent risk factor for prognosis of left-sided colon cancers. RAH is an appropriate operation method for left-sided colon cancer, with perioperative and long-term outcomes comparable to those of laparoscopy. Meanwhile, RHA has longer operative time and higher cost.

## Introduction

Colon cancer is one of the most common malignant tumors and at the forefront of tumor mortalities worldwide [1]. Surgical resection is the main curative method for colon cancer. Resection methods that minimize pain and ensure faster recovery have been investigated. Consequently, colon cancer treatment has made great progress, from open surgery to minimally invasive approach. Laparoscopic surgery is a minimally invasive technique widely applied in abdominal surgery. However, conventional laparoscopy has some limitations, such as two-dimensional imaging, a steep learning curve, amplified physiological tremor, restricted range of motion, and ergonomic discomfort. Recently, the use of robots to overcome these shortcomings has been employed as a new strategy for treating colon cancers in the era of minimally invasive surgery. Robotic surgical systems have some advantages, such as a three-dimensional surgical view and increased dexterity and steadiness, which ensure efficiency in surgery and thus benefit patients [2]. As a result, robotic surgery has attracted increasing attention from surgeons. Previous studies have demonstrated that robot-assisted surgery is a safe and effective way to perform colon cancer resection, and it is superior to laparoscopic surgery in multiple aspects, such as less blood loss and a lower conversion rate to open surgery [3]. However, a few studies have focused on the surgical and prognostic outcomes of left hemicolectomy in specialty [4]. This study aimed to compare the perioperative and long-term outcomes of robot-assisted hemicolectomy (RAH) versus laparoscopy-assisted hemicolectomy (LAH) for left-sided colon cancers.

## Materials and methods

This study comprised patients who underwent RAH or LAH for left-sided colon cancer in the Chinese PLA General Hospital from January 2012 to December 2018. The inclusion criteria were malignant left-sided colon tumors confirmed by a preoperative or postoperative pathology report, patients who underwent a left hemicolectomy with curative intent, and those who had undergone a minimally invasive procedure, the robot- or laparoscopy-assisted surgery. Patients with benign colonic diseases, recurrent cancers, and metastatic tumors that invaded other organs were excluded. To compare the long-term efficacy of the two surgical approaches, patients with severe cardiovascular or cerebrovascular diseases and those who underwent neoadjuvant chemotherapy before surgery were not included. All surgeries were performed in the same institution, and D3 radical surgery was conducted according to the Japanese Society for Cancer of the Colon and Rectum guidelines [5]. All surgeons had equivalent clinical qualifications. RAH was performed using the da Vinci Si Surgical System (Intuitive Surgical, Inc., Sunnyvale, USA).

The following information was collected retrospectively: (1) baseline data of the two groups: sex; age; body mass index (BMI); American Society of Anesthesiologists (ASA) classification [6], characteristics of tumors, such as location, size, differentiation, pathological stage, and type (the pathological staging was based on the National Comprehensive Cancer Network Guidelines Colon Cancer, Version 4. 2019 [7]); (2) perioperative indicators: operative time; estimated blood loss; intraoperative blood transfusion; number of retrieved lymph nodes; postoperative recovery data, such as days to bowel recovery, initiation of liquid diet, and duration of gastric tube, urine tube, and abdominal drainage tube; and hospitalization expenses; and (3) postoperative complications, evaluated by the Clavien–Dindo classification [8]: fever, pulmonary infection, anastomotic leakage, bleeding, incomplete ileus, gastrointestinal dysfunction (gastroplegia and enteroplegia), and postoperative Intensive-Care Unit (ICU) stay.

Designated doctors managed patient follow-ups, which were conducted quarterly in the first year, semiannually in the next 2 years, and annually thereafter. Information regarding the elapsed time, metastasis, and survival status was collected. This project was approved by the Ethics Committee of the Chinese PLA General Hospital.

### Statistical analysis

Statistical analyses were conducted using SPSS 24.0 (Statistic Package for Social Science; SPSS Inc., Chicago, IL, USA). Categorical variables were compared using the standard Chi-square test. Continuous variables, shown as the mean ± standard deviation, were compared using Student’s *t* test and data shown as median (upper quartile, lower quartile), were analyzed by Mann–Whitney *U* test, if the *p* value in the Kolmogorov–Smirnov test of normality was lower than 0.05.

All patients were followed up until death or until the last follow-up date of April 30, 2020. The Kaplan–Meier method was used to draft the survival curves, and the log-rank test was used to compare the differences. Multivariate analyses for survival were performed using the Cox proportional hazard model. A *p* value less than 0.05 was considered statistically significant.

## Results

**Fig. 1:**
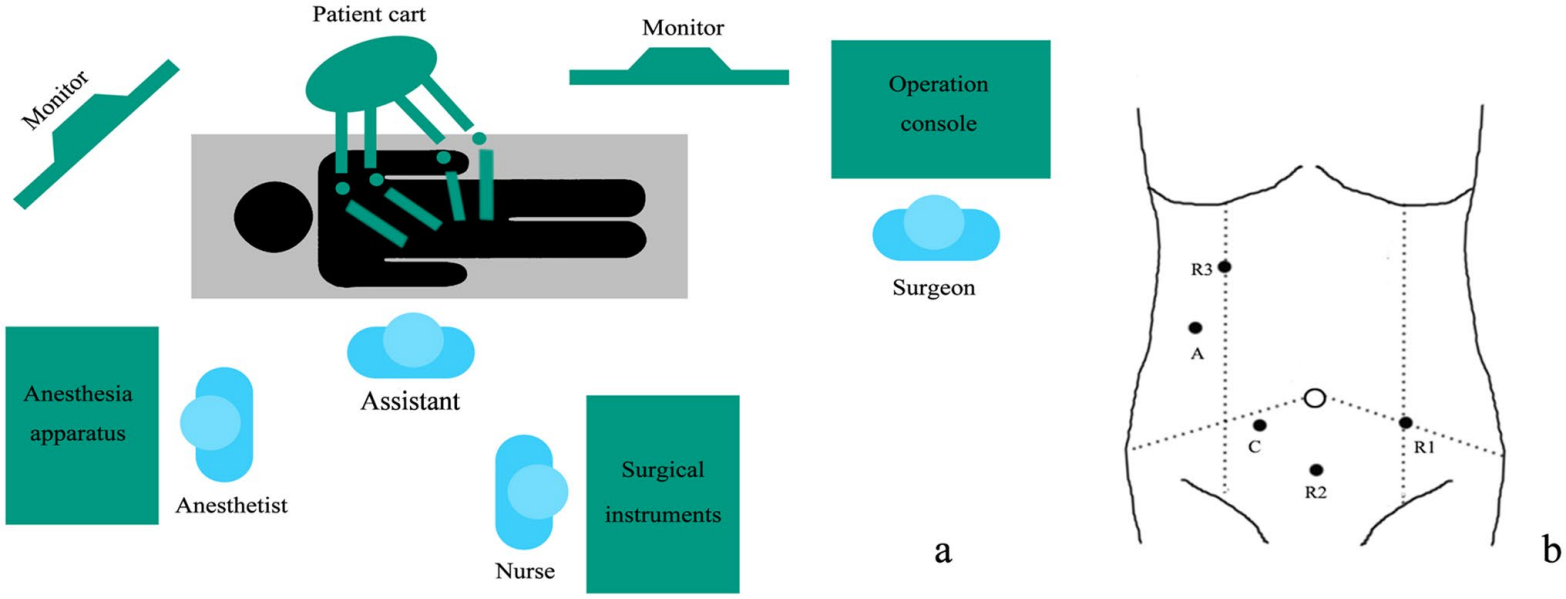
Operating room setup and trocars location of robot-assisted left hemicolectomy. **a** Operating room setup. **b** Trocars location *C* camera; *R1-3* robotic instrument; *A* assistant

A total of 460 patients were included (RAH, *n* = 205; LAH, *n* = 255). All surgeries were performed successfully with no conversions to open surgery. Figure 1 shows the operating room setup and trocars location for the robot-assisted left hemicolectomy.

### Baseline data

The baseline information of the two groups was comparable (Table 1). The mean patient age was 60.35 ± 11.33 years in the RAH group and 60.36 ± 11.04 years in the LAH group. There were no significant differences in the clinical characteristics, such as sex, BMI, and ASA classification. Data for preoperative tumor markers, carcinoembryonic antigen (CEA), and carbohydrate antigen 19–9 (CA19-9) were included in this study. The data are shown as median (upper quartile, lower quartile) and there were no significantly statistical differences between the two groups. All surgeries achieved negative resection margins and the pathology results in each group were comparable. The tumor sizes of the patients were similar at 4.08 ± 1.63 mm in the RAH and 4.14 ± 1.83 mm in the LAH group. The pathological stage of I, II, and III were 20.49%, 46.83%, and 32.68% in RAH group, and 18.82%, 47.06%, and 34.12% in LAH group, respectively, and there was no statistical difference. The location, differentiation, type of tumor, and the occurrence of intravascular cancer embolus, nerve invasion, and tumor nodules showed no significant difference.

**Table 1:** Characteristics of the patients and pathology results of the two groups

Variable	RAH (*n* = 205)	LAH (*n* = 255)	*p*
Age (year)	60.36 ± 11.33	60.36 ± 11.04	0.993
Gender (%)			0.139
Male	123 (60.00)	170 (66.67)	
Female	82 (40.00)	85 (33.33)	
BMI (kg/m^2^)	24.80 ± 3.34	24.78 ± 3.16	0.948
ASA scores (%)			0.533
I	6 (2.93)	8 (3.14)	
II	173 (84.39)	223 (91.37)	
III	26 (12.68)	24 (9.41)	
CEA	3.05 (1.95,7.84)	3.35 (2.06,8.00)	0.532
CA19-9	12.65 (7.41,23.03)	12.41 (7.74,22.08)	0.890
Tumor location (%)			0.200
Descending colon	33 (16.10)	53 (20.78)	
Sigmoid colon	172 (83.90)	202 (79.22)	
Tumor size (mm)	4.08 ± 1.63	4.14 ± 1.83	0.729
Pathological stage (%)			0.890
I	42 (20.49)	48 (18.82)	
II	96 (46.83)	120 (47.06)	
III	67 (32.68)	87 (34.12)	
Tumor differentiation (%)			0.391
Well differentiation	14 (6.83)	26 (10.20)	
Moderate differentiation	173 (84.39)	204 (80.00)	
Poor differentiation	18 (8.78)	25 (9.80)	
Pathological type (%)			0.665
Ulcerative type	138 (67.32)	168 (65.88)	
Polypoid type	40 (19.51)	46 (18.04)	
Other type	27 (13.17)	41 (16.08)	
Intravascular cancer embolus (%)	16 (7.80)	21 (8.24)	0.866
Nerve invasion (%)	7 (7.80)	15 (5.88)	0.218
Tumor nodules (%)	12 (5.85)	18 (7.06)	0.603

*RAH* robot-assisted hemicolectomy; *LAH* laparoscopy-assisted hemicolectomy; *BMI* body mass index; *ASA* American Society of Anesthesiologists; *CEA* carcinoembryonic antigen; *CA19-9* carbohydrate antigen 19–9

### Perioperative outcomes

Table 2 shows the perioperative surgical outcomes in each group. The operative time in the RAH group (150.23 ± 43.77 min) was significantly longer than that in the LAH group (125.85 ± 38.67 min) (*p* < 0.001). There were significant differences in surgery costs (6.33 ± 1.50 vs. 2.88 ± 0.72 thousand $, *p* < 0.001) and total hospital costs (14.97 ± 3.05 vs. 9.05 ± 2.31 thousand $, *p* < 0.001) between the RAH and LAH groups.

**Table 2:** Perioperative surgical outcomes of the two groups

Variable	RAH (*n* = 205)	LAH (*n* = 255)	*p*
Operative time (min)	150.23 ± 43.77	125.85 ± 38.67	< 0.001
Estimated blood loss (ml)	84.54 ± 69.81	88.27 ± 66.87	0.559
Intraoperative blood transfusion (%)	7 (3.41)	12 (4.71)	0.489
Number of retrieved lymph nodes (*n*)	14.37 ± 4.64	14.33 ± 5.23	0.944
Days of bowel recovery (d)	3.63 ± 1.15	3.67 ± 1.19	0.707
Initiation of liquid diet (d)	4.48 ± 1.62	4.52 ± 1.54	0.814
Duration of gastric tube (d)	2.89 ± 1.63	2.86 ± 1.45	0.840
Duration of urine tube (d)	5.35 ± 2.53	4.94 ± 2.09	0.058
Duration of abdominal drainage tube (d)	8.05 ± 2.30	7.83 ± 1.89	0.267
Postoperative hospital stay	9.23 ± 3.37	9.14 ± 3.08	0.758
Surgery costs ($, *10^3^)	6.33 ± 1.50	2.88 ± 0.72	< 0.001
Total hospital costs ($, *10^3^)	14.97 ± 3.05	9.05 ± 2.31	< 0.001
Postoperative chemotherapy (%)			0.568
XELOX regimen	53 (25.85)	68 (26.67)	
Other regimen	37 (18.05)	55 (21.57)	
No chemotherapy	115 (56.10)	132 (51.76)	
Postoperative complication (%)			0.732
Fever	11 (5.37)	16 (6.27)	
Pulmonary infection	6 (2.93)	5 (1.96)	
Anastomotic leakage	6 (2.93)	8 (3.14)	
Bleeding	7 (3.41)	11 (4.31)	
Incomplete ileus	8 (3.90)	4 (1.57)	
Gastrointestinal dysfunction	9 (4.39)	6 (2.35)	
Postoperative ICU stay	7 (3.41)	9 (3.53)	
Clavien–Dindo classification (%)			0.676
I	11 (5.37)	16 (6.27)	
II	30 (14.63)	26 (10.20)	
III	6 (2.93)	8 (3.14)	
IV	7 (3.41)	9 (3.53)	
V	0	0	

*RAH* robot-assisted hemicolectomy; *LAH* laparoscopy-assisted hemicolectomy; *ICU* intensive-care unit

The estimated blood loss, intraoperative blood transfusion, and number of retrieved lymph nodes were comparable. Indicators of postoperative recovery, such as days of bowel recovery, initiation of liquid diet, and duration of gastric tube, urine tube, and abdominal drainage tube, showed no statistical difference. The postoperative hospital stay was comparable (9.23 ± 3.37 days vs. 9.14 ± 3.08 days, *p* > 0.05).

Each patient at pathological stage II with risk factors such as intravascular cancer embolus, nerve invasion, and perforation in tumor site, etc., or more advanced stage was recommended for adjuvant chemotherapy. XELOX (Xeloda + Oxaliplatin) were the most commonly applied regimen, and there was no significant difference in adjuvant chemotherapy between the two groups. No significant differences were observed in terms of postoperative complications, including fever, pulmonary infection, anastomotic leakage, bleeding, incomplete ileus, gastrointestinal dysfunction, and postoperative ICU stay.

### Long-term outcomes

**Fig. 2:**
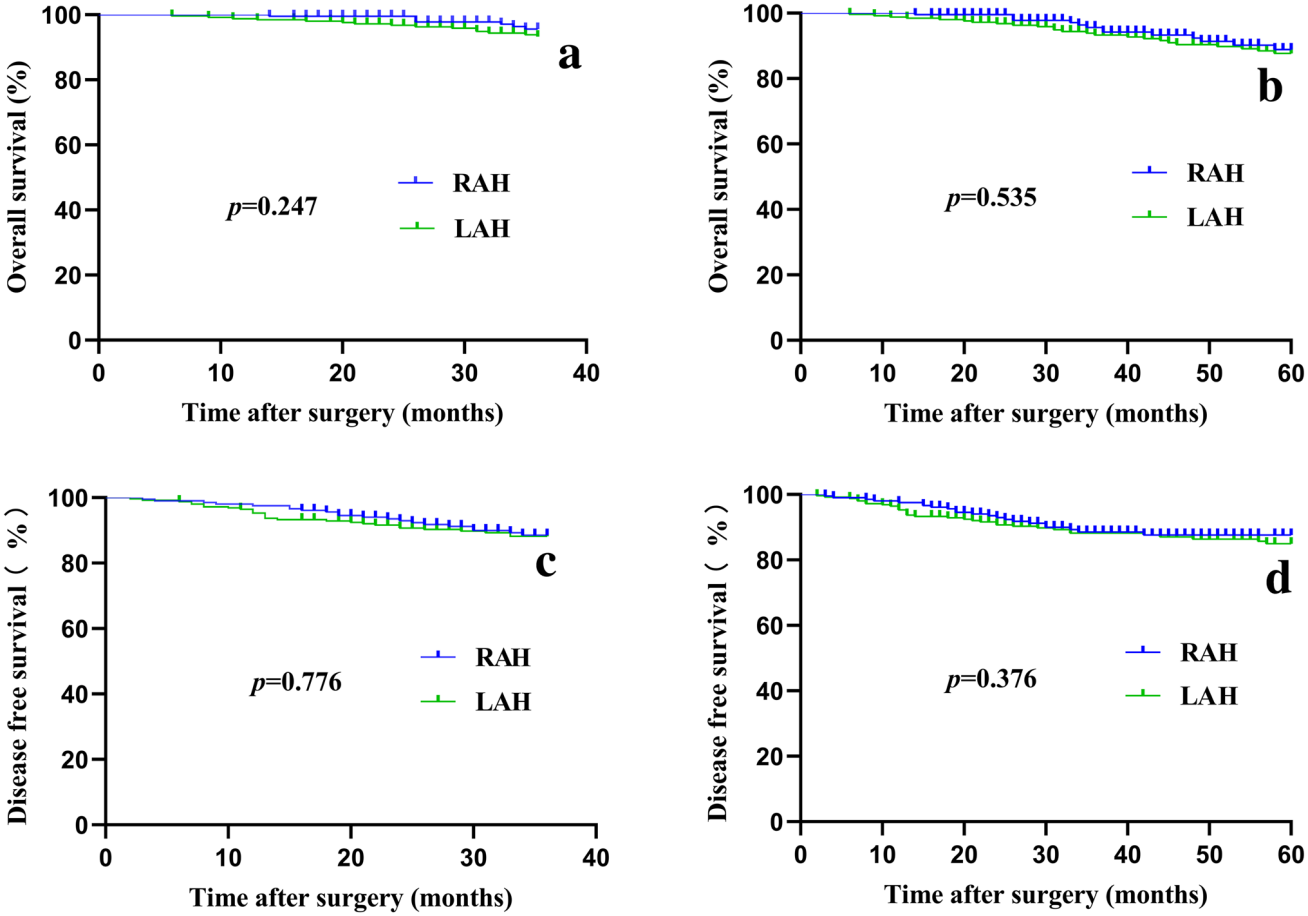
The overall survival (OS) and disease-free survival (DFS) of the two groups. **a** The 3-year OS. **b** The 3-year DFS. **c** The 5-year OS. **d** The 5-year DFS. *RAH* robot-assisted hemicolectomy, *LAH* laparoscopy-assisted hemicolectomy

**Fig. 3:**
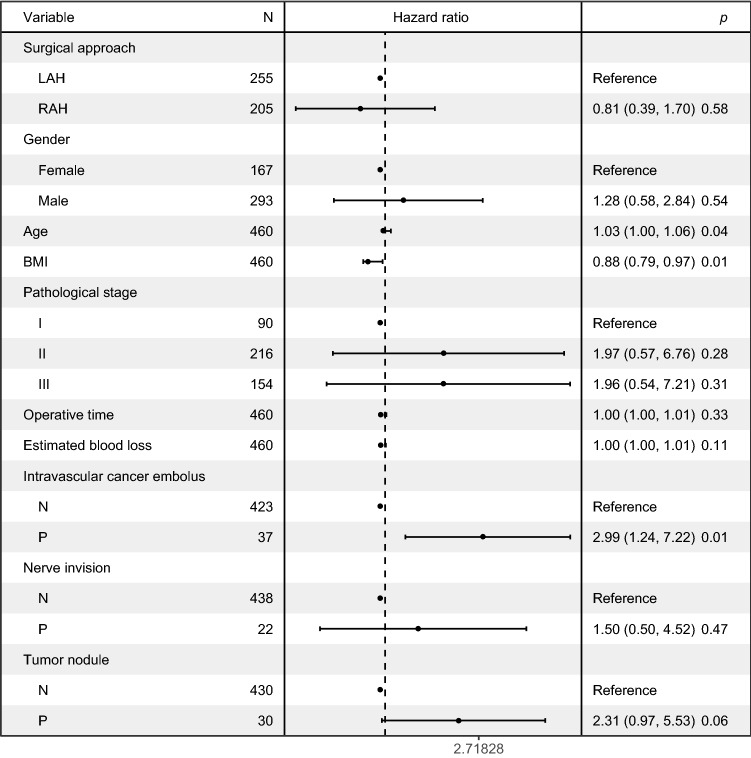
The Cox proportional hazard model for multivariate survival analyses. *RAH* robot-assisted hemicolectomy, *LAH* laparoscopy-assisted hemicolectomy, *BMI* body mass index, *N* negative, *P* positive

The follow-up period was 48.64 ± 22.40 months in the RAH group and 55.91 ± 26.03 months in the LAH group (*p* = 0.002) (Table 3). The reason for this difference was that more patients chose laparoscopy-assisted surgery in the early stage of this study, while robot-assisted surgery saw an increase in popularity more recently. The 3-year overall survival (OS) rate was 96.59% vs. 94.12%, and the 5-year OS rate was 93.17% vs. 90.59% in the RAH and LAH groups, respectively (*p* > 0.05 for both). The 3-year disease-free survival (DFS) rate was 89.76% vs. 89.02%, and the 5-year DFS rate was 89.27% vs. 87.06%, respectively (*p* > 0.05 for both). Kaplan–Meier curves for OS and DFS showed no significant differences between the two groups (Fig. 2). Age, BMI, and intravascular cancer embolus were independent risk factors for OS in multivariate analyses (Fig. 3). Moreover, the robotic approach was not an independent risk factor for OS.

**Table 3:** Long-term outcomes of the two groups

Variable	RAH (*n* = 205)	LAH (*n* = 255)	*p*
Months of follow-up	48.64 ± 22.40	55.91 ± 26.03	0.002
DFS (%)			
3 years	89.76	89.02	0.776
5 years	89.27	87.06	0.376
OS (%)			
3 years	96.59	94.12	0.247
5 years	93.17	90.59	0.535

*RAH* robot-assisted hemicolectomy; *LAH* laparoscopy-assisted hemicolectomy; *DFS* disease-free survival; *OS* overall survival

## Discussion

The comparison of surgical effects between robot and laparoscopy in digestive diseases is a hot topic, while studies on the outcomes of robot-assisted tumor resection of left-sided colon were relatively few. Some studies on benign lesions, such as the diverticulum, also showed the advantages and disadvantages of the two surgical procedures. Grass et al. reported that robotic resection was feasible and safe in left-sided complicated diverticular disease [9] and Crolla et al. reported that sigmoid and rectal surgery of T4 stage tumors with multivisceral resection can be achieved with the robot-assisted method [10]. Bastawrous et al. from the United States collected data of 13,240 sigmoidectomies and concluded that laparoscopy had a higher conversion rate than robot (13.6% vs. 8.3%) [11]. Alharthi et al. analyzed the data of 197,053 patients who underwent a sigmoidectomy, and reported that the robotic approach had shorter hospital stay and higher total hospital charges than laparoscopic surgery [12]. A study from Korea described that the mean operative time was longer in robotic left colectomy than in the laparoscopy with complete mesocolectomy of 73 patients, while the robot enabled dexterous dissection for the multi-directional pathway during left mesocolic mobilization [13]. Dwyer et al. supported the potential benefits of synchronous robotic liver resection in colon cancer as this method demonstrated low blood loss (150–1000 mL), appropriate length of hospital stay (3–10 days), and no 30-day mortality [14].

In our study, both the perioperative and long-term outcomes were comparable between the RAH and LAH groups. The operative time was longer and the medical cost was higher in the RAH group than in the LAH group, which, to a large extent, was consistent with the findings of previous reports. The increase in operative time was mainly due to the installation of the robot arms and the placement of the trocars, which may decrease with improved performance proficiency and tacit order of teamwork. The major shortcoming of robotic surgery was the significant increase in medical cost compared with that of laparoscopy. The cost of robots mainly comes from high selling price, expensive consumables, and daily maintenance expense. The emergence of surgical robots from multiple manufacturers, such as MicroHand S from China and Senhance robotic system from the United States, has resulted in the introduction of robots in the clinical settings with good results [15–18]. With increasing competition, the cost of robotic surgery is expected to decrease, which may ultimately promote their application and bring benefits to patients.

Despite the disadvantages of longer operative times and higher cost, the robotic surgical system has some advantages, such as enlarged three-dimensional views, allowing flexibility of wrist movement, and filtering hand tremors, which allow surgeons to perform meticulous operations in a small space and make it convenient to deal with intraoperative emergencies such as bleeding. Besides, some studies have shown that robots conferred superior ergonomic benefits and reduced workloads for surgeons when compared to laparoscopy as well as can possibly optimize surgeon performance by reducing fatigue [19, 20].

Another advantage is the short learning curve for surgeons. Symer et al. researched a total of 2763 procedures for robotic colorectal resection [21]. They reported that after surgeons completed their first 27 cases, a decrease in iatrogenic complications was observed, and this trend continued as the case volume increased. Gerbaud et al. further reported that the transition from laparoscopic to robot-assisted colectomy with intracorporeal anastomosis may not entail any increase in the morbidity rate or reduce the oncologic quality of the surgery when performed by a surgeon with experience in laparoscopic surgery [22].

Overall, the robotic surgical system may have some technical advantages when compared with the conventional laparoscopy which ultimately benefited the patients. Consistent with previous studies, this retrospective study showed that RAH had comparable perioperative and long-term outcomes with laparoscopic surgery. Meanwhile, robot had longer operative time and higher cost. Furthermore, the emergence of surgical robots produced by multiple manufacturers will have improved performance and reduce patients’ burden of healthcare cost, and robotic surgery may have a good prospect in clinic.

## Data Availability

All the data were shown in the study.
